# RETRACTION: The Effect of *Leonurus sibiricus* Plant Extracts on Stimulating Repair and Protective Activity against Oxidative DNA Damage in CHO Cells and Content of Phenolic Compounds

**DOI:** 10.1155/omcl/9828430

**Published:** 2026-07-30

**Authors:** Oxidative Medicine and Cellular Longevity

RETRACTION: P. Sitarek, E. Skała, H. Wysokińska, et al., “The Effect of *Leonurus sibiricus* Plant Extracts on Stimulating Repair and Protective Activity against Oxidative DNA Damage in CHO Cells and Content of Phenolic Compounds,” *Oxidative Medicine and Cellular Longevity*, 2016, 5738193, https://doi.org/10.1155/2016/5738193.

The above article, published online on 14 December 2015 in Wiley Online Library (wileyonlinelibrary.com), has been retracted by agreement between the Journal Editor‐in‐Chief, Jeannette Vasquez Vivar; and John Wiley & Sons Ltd. The retraction has been agreed due to concerns that have been raised with Figure 4c, also reported on PubPeer [1] (but noted incorrectly as Figure 5c). Specifically, there are multiple instances of identical bands shown in Figure 4c, reported as representing both the same and different conditions.

The authors responded to the concerns; however, due to the time elapsed since the study, they could not provide the original images for this figure. After assessing the response to the concerns, we conclude that neither the results nor the conclusions can be considered reliable, and the article is therefore retracted.

The authors disagree with the retraction.
